# AI-Assisted Molecular Biosensors: Design Strategies for Wearable and Real-Time Monitoring

**DOI:** 10.3390/ijms27073305

**Published:** 2026-04-06

**Authors:** Sishi Zhu, Jie Zhang, Xuming He, Lijun Ding, Xiao Luo, Weijia Wen

**Affiliations:** 1 Thrust of Advanced Materials, The Hong Kong University of Science and Technology (Guangzhou), Guangzhou 511400, China; szhu260@connect.hkust-gz.edu.cn (S.Z.);; 2 Department of Data and Systems Engineering, The University of Hong Kong, Hong Kong 999077, China; 3 Department of Physics, The Hong Kong University of Science and Technology, Hong Kong 999077, China

**Keywords:** biosensor, wearable, AI, real-time monitoring, molecular detection

## Abstract

Artificial intelligence (AI) has become a transformative tool in the field of molecular biosensing, enabling data-driven optimization in sensor design, signal processing, and real-time monitoring. AI promotes the discovery of biomarkers, the design of high-affinity receptors, and the rational engineering of sensing materials, thereby enhancing sensitivity, specificity, and detection accuracy. In the development of biosensors, AI-assisted strategies have accelerated the identification of novel molecular targets, guided the design of proteins and aptamers with enhanced binding performance, and optimized plasmonic and nanophotonic structures through forward prediction and inverse design frameworks. The integration of artificial intelligence has significantly enhanced the performance of various biosensing platforms, including optical, electrochemical, and microfluidic biosensors. It also enabled automatic feature extraction, noise reduction, dimensionality reduction, and multimodal data fusion, overcoming the challenges posed by complex signals, environmental interference, and device variations. These capabilities are particularly crucial for wearable molecular biosensors, as low signal strength, motion artifacts, and fluctuations in physiological conditions impose strict requirements on robustness and real-time reliability. This review systematically summarizes the latest advancements in AI-assisted molecular biosensors, highlighting representative sensing strategies and algorithms for wearable and real-time monitoring, and discusses the current challenges and future development opportunities of intelligent biosensing technologies.

## 1. Introduction

In recent years, wearable biosensors have attracted widespread attention due to their ability to collect non-invasive, continuous, and real-time physiological information [1]. Traditional biological detection usually relies on laboratory analysis equipment, which is complex to operate and has a long detection cycle. It is also difficult to meet the dynamic monitoring requirements of individualized health management. In contrast, wearable biosensors, which adhere to the human skin or embedded within wearable products or textiles, enable real-time monitoring of multiple sources of information such as urine [2,3], sweat [4,5], interstitial fluid [6], blood [7,8,9], and physiological signals [10,11]. They can achieve continuous assessment of individual health status, providing important technical support for early disease diagnosis, chronic disease management, and sports physiology analysis. Breakthroughs in flexible electronic materials and micro- and nano-manufacturing technologies have significantly advanced the development of wearable sensors. Sensors based on flexible polymers, nanocomposites, and micro-structural designs not only possess excellent mechanical flexibility properties but can also maintain stable signal output in complex dynamic environments. Moreover, the emergence of multimodal sensing strategies enables a single device to simultaneously monitor multiple physiological indicators, such as biochemical molecules, pressure, temperature, and electrophysiological signals, thereby significantly enhancing the information dimension and diagnostic accuracy of health monitoring systems.

However, signals acquired from wearable devices are inherently affected by motion artifacts [12], environmental fluctuations [13], high power usage [14], and long-term sensor drift [15]. In non-invasive samples, concentrations are typically low and temporally variable, further complicating reliable molecular detection. Traditional calibration strategies and fixed-threshold analysis are often insufficient to deal with these complexities. By enabling adaptive signal interpretation and data-driven correction, AI provides the computational foundation required for stable, continuous, and personalized molecular monitoring in wearable formats. Under such conditions, AI plays an increasingly important role in molecular sensors. Several classic AI algorithms provide the computational foundation required for stable, continuous, and personalized molecular monitoring in wearable formats by virtue of their robust data analysis capabilities, and these algorithms can be mainly categorized into machine learning and deep learning. Table 1 presents several classic AI algorithms. Leveraging their robust data analysis and prediction capabilities, these algorithms provide the computational foundation required for stable, accurate, and personalized molecular biosensing.

AI serves as a powerful tool for optimizing biosensors, gaining considerable interest [22,23]. Algorithms’ ability to efficiently learn from and predict complex datasets can be applied to mining potential biomarkers from massive amounts of bioinformatics and clinical data, overcoming the limitations of traditional experimental screening methods [24,25]. Moreover, machine learning models can predict the binding affinity between recognition elements and target biomarkers, guiding the rational design of recognition elements such as aptamers and antibodies, significantly shortening the research and development cycle [26,27]. For developing advanced sensing materials, artificial intelligence can simulate the structure–activity relationships of sensor-sensitive materials, cost-effectively guiding the design and synthesis of functional materials with high sensitivity and stability [28,29]. Furthermore, AI’s robust data processing capabilities—including feature extraction, noise reduction, rapid identification, regression, and clustering—optimize various biosensor performances in signal amplification, noise interference mitigation, accurate multi-target detection, and real-time monitoring [30,31,32]. In summary, AI-assisted optimization of biomolecular sensors manifests in improved initial biosensor design, enhanced performance across various platforms, and accelerated real-time monitoring applications.

**Figure 1:**
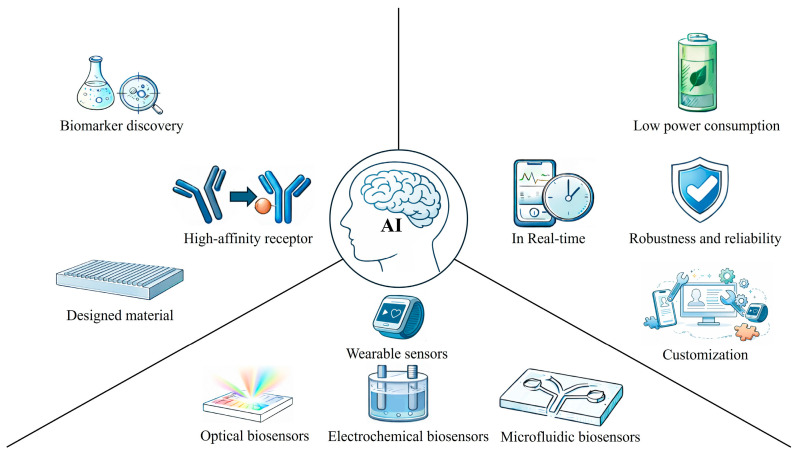
AI and biosensor integration network.

Although significant progress has been made in wearable biosensors in recent years, there are still many challenges in terms of long-term stability, biocompatibility, energy management, and data security. Therefore, in this review (Figure 1), we will mainly discuss the AI-assisted biosensor strategy in detecting molecules and hot spots in wearable sensors. We hope this review gives ideas for developing highly sensitive, multi-functional, integrated, and intelligent data processing methods, which are of great significance for promoting the wide application of wearable biosensing technology in clinical medicine and health management.

## 2. AI-Powered Biosensor Design

Molecular sensors, as an analytical tool characterized by high sensitivity, high specificity, miniaturization, and rapid response, are of great significance for disease monitoring, clinical diagnosis, and drug development. Basically, the molecular biosensing process involves biological targets, biological receptors, and transducers. The specific binding of small-molecule targets to receptors generates biochemical signals, which are then converted by transducers into readable signals. AI holds the potential to drive innovation in molecular biosensing by enhancing iterative efficiency through big data-driven training and efficient prediction from a design perspective. Recent research has focused on identifying novel biomarkers within vast, complex biomolecular landscapes to expand the range of diagnosable diseases, improve detection accuracy, and advance personalized medicine. AI has revolutionized the analysis of complex gene databases, enabling precise indicator screening by reducing noise in single-omics data and uncovering nonlinear correlations through multi-omics data integration. This accelerates the discovery of novel biomarkers and elucidates underlying mechanisms.

### 2.1. New Biomarker Discovery

**Figure 2:**
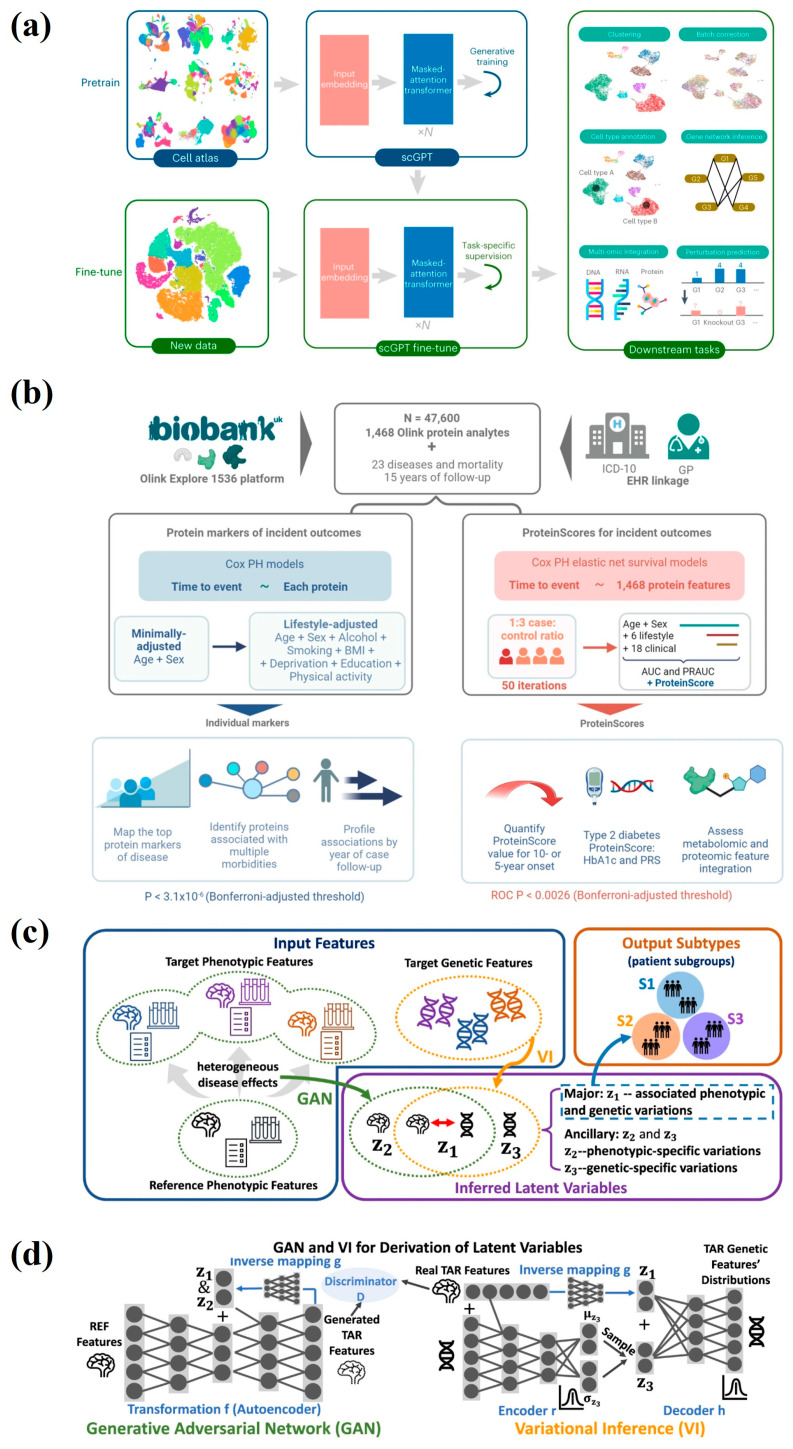
New biomarkers discovered by AI. (**a**) A flow chart of scGPT presents downstream tasks based on a fine-tuned model. Images reprinted from [37] with permission. (**b**) Associations between protein analytes and multiple morbidities were explored by Cox PH models and Cox PH elastic net survival models. Images reprinted from [38] with permission. (**c**) Schematic diagram of Gene-SGAN, which identifies patient subgroups by genetic and phenotypic features. (**d**) GAN and VI framework estimates genetic feature distribution. Images reprinted from [40] with permission.

Biomarkers refer to measurable indicators in biological samples such as blood, bodily fluids, and tissues that reflect an individual’s health status, screen for diseases, and monitor treatment processes [33]. Biomarkers can be categorized into four types: molecular markers (nucleic acids, proteins, metabolites, etc.); histological markers; imaging markers (computed tomography scans, magnetic resonance imaging, or positron emission tomography scans, etc.); and physiological markers (blood pressure, heart rate, blood glucose, etc.) [34]. As biomarker databases continue to expand, AI algorithms have become the core driver for handling vast datasets, significantly accelerating the speed and enhancing the accuracy of biomarker discovery [35]. For single data types, AI algorithms offer numerous capabilities, including batch correction, type clustering, feature selection, dimensionality reduction, and integration of different data types within the same modality [36]. Cui et al. [37] reported a generative AI-based model, scGPT, pretraining on large-scale single-cell sequencing data. Through self-supervised learning, the model perceives genes as “words” and encoded proteins as “text,” thereby comprehending key biological information linking genes to cells. During the fine-tuning stage, multiple critical tasks—including cell type annotation, multimodal integration, perturbation response prediction, and gene network inference—were optimized for implementation (Figure 2a). For single-cell multi-omic data, scGPT demonstrated superior cell type clustering under both paired and mosaic integration settings compared to established integration models. Gadd et al. [38] reported ProteinScores, a model based on cox proportional hazards regression and elastic net, offering insight into protein biomarker discovery and incident disease prediction (Figure 2b). They train with large-scale proteomic data provided by the UK Biobank, 1468 plasma protein information from 47,600 people. ProteinScores shows better performance in predicting age-related disease risks than a comprehensive set with 26 covariates, particularly for type 2 diabetes.

In multimodal analysis, common deep learning algorithms fuse different data types [39]. Yang et al. [40] reported Gene-SGAN, a weakly-supervised deep clustering method, linked MRI and single-nucleotide polymorphism data to identify disease subtypes and endophenotype (Figure 2c). The multimodal deep learning framework maps brain imaging features from healthy controls to patient cohorts, simulating disease-induced structural alterations. Subsequently, within the latent space generated by GANs, the model employs Variational Inference to estimate the distribution of genetic features, thereby establishing associations between imaging changes and genetic variations (Figure 2d). Four dementia—related subtypes and five clinically distinct hypertension-related subtypes are identified by GeneSGAN with reproducible imaging signatures, genetic architectures, and clinical profiles. Spratt et al. [41] reported a multimodal predictive model, combining pathology and clinical data to guide androgen deprivation therapy (ADT) with radiotherapy. According to advice from model calculation, patients with prostate cancer were treated with at least 4-month ADT along with radiotherapy. The follow-up patients’ status identified that adding ADT to radiotherapy led to reduced distant metastasis.

### 2.2. High-Affinity Receptor

Designing high-affinity receptors (including proteins, aptamers, peptides, etc.) is crucial for enhancing the sensitivity and specificity of molecular sensors [42]. Leveraging machine learning and deep learning algorithms enables efficient exploration of interaction patterns between receptors and target molecules, enabling precise prediction of binding sites and affinity potential [43,44]. This provides reliable predictions for the targeted design of high-affinity receptors, significantly boosting design efficiency and success rates. An et al. [45] designed pseudocycle scaffolds with high shape complementarity to molecules by AlphaFold2 and the ProteinMPNN deep learning model for downstream sensing. Through two-round binder screening, the highest affinity binder for CHD was verified as 4.7 nM. The integration of the CHD binder and nanopore lowered the noise and considerably increased the closed-state conductance duration, leading to a lower frequency required for sensing. Torres et al. [46] reported a deep learning based strategy, the Hallucination and RFdiffusion model, designing picomolar-affinity protein binders of helical peptides. The Hallucination method requires no information on the bound structure for binder design, while the RFdiffusion method is more computationally efficient in binder affinity and specific to helical targets. In addition, RFdiffusion model enables de novo binder design for clinical peptides, which is demonstrated in parathyroid hormone sensitive detection with 10 nM LoD.

Similar to monoclonal antibodies, aptamers are a class of chemically synthesized short single-stranded nucleic acids, typically ranging from 20 to 200 nt in length, that specifically bind to target molecules such as peptides, proteins, and cells. Machine learning and deep learning presented great potential in aptamer affinity prediction, thus assisting aptamer design in a convenient and low-cost way [47,48]. Amu et al. [49] designed a modified aptamer using a machine learning algorithm, predicting high-affinity modification and elucidating underlying mechanisms. The stacking model was based on Random Forest, Extreme Gradient Boosting, and Light Gradient Boosting Machine, training with 422 modified aptamer sequences and displaying a high accuracy with a 0.82 correlation coefficient. In-depth interpretation of the model showed that the enhanced affinity after modification was attributed to newly formed hydrophobic interactions and more stable aptamer structures.

### 2.3. Designed Material

**Figure 3:**
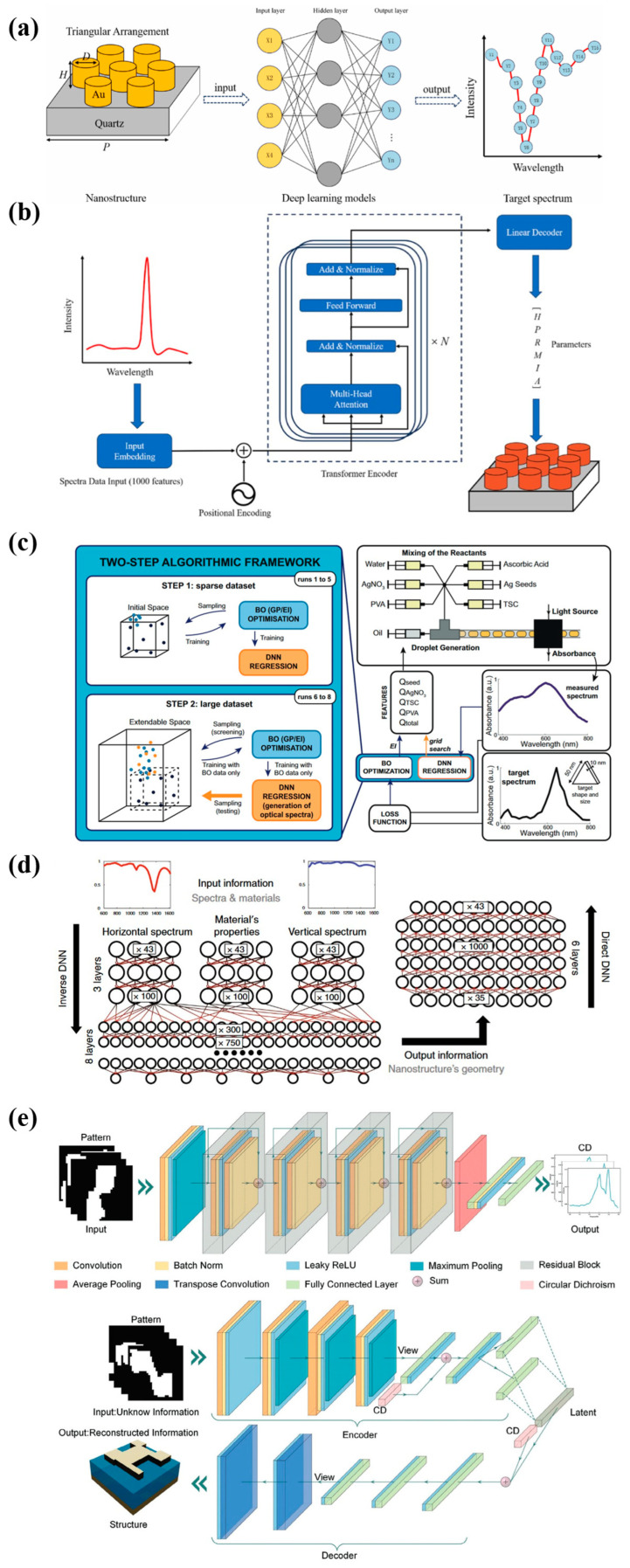
Material design guided by AI. (**a**) The forward network predicts the target spectrum. (**b**) The reverse network for structure predictions. Images reprinted from [58] with permission. (**c**) A two-step framework for desired nanoparticles at a microfluidic producing platform. Images reprinted from [59] with permission. (**d**) Deep neural network architecture. Images reprinted from [60] with permission. (**e**) Forward and inverse network design. Images reprinted from [61] with permission.

The excitation of free electron oscillations on the surface of metal nanostructures by light is termed plasmonic resonance, which is sensitive to nanoparticle size, shape, and surrounding medium [50]. The interaction between nanostructures and biomolecules can be converted into optical signals through refractive index changes, plasmonic coupling effects, and surface-enhanced spectroscopy, enabling ultra-sensitive, label-free, and real-time detection [51,52]. Typically, plasmonic nanostructure design is an iterative process, progressing from designing multiple structures, precision fabrication, and property characterization to application [53]. With the establishment of complex databases and the maturation of algorithmic techniques, machine learning and deep learning have accelerated the optimization steps in nanoplasmonics, enhancing iteration efficiency [54,55,56]. AI has gained great attention in rational design, predicting plasmonic properties from nanostructure or synthesis conditions [57]. Liu et al. [58] constructed a spectral parameter multi-layer perception neural network to guide metasurface design and predict nanophotonic biosensor performance. Traditional photonics simulation is replaced by an algorithm, mapping structural features to optical responses in a rational and time-saving way. The forward network provides spectral data and then extracts sensing parameters (Figure 3a). The inverse network (Figure 3b), Transformer, outputs the resulting parameters, which are tested by the High Q and Low FWHM algorithm to satisfy the sensor design specifications. Through hundreds of iterations, the customized surface lattice resonance biosensor is designed with an accurate deep learning-based prediction method. Additionally, deep learning provides guidance for synthesizing nanomaterials with ideal optical properties. For example, a deep neural network combined with a Gaussian process-based Bayesian optimization screened the sparse dataset, drove microfluidic high-throughput experimental platforms, and converged to the desired silver nanoparticle synthesis [59] (Figure 3c). Through the Shapley Additive Explanations feature importance and matrix analysis, it was revealed that silver nitrate concentration and seed flow rate are key factors influencing spectral shape and amplitude, indicating model interpretability.

Also, the nanostructure’s geometry prediction from the optical spectrum has been accurately achieved by deep learning. Malkiel et al. [60] reported a bidirectional network, Geometry-predicting-network (GPN) training, and Spectrum-predicting-network (SPN). The inverse problem task, predicting the geometry by a desired far-field spectrum input, was prohibitive due to the high nonlinearity. Thus, network architectures with different depths were trained, in which three parallel group layers followed by eight fully connected join layers showed significant performance (Figure 3d). Compared to time-consuming nanostructure design based on standard simulation tools, GPN solved the task in seconds. Similarly, Zhang et al. [61] designed novel chiral metasurfaces in assistance of a deep learning algorithm for the exceptional circular dichroism (CD) signal. The globe design framework for selecting practical and high-quality patterns integrated forward screen network based on the deep residual network ResNet18 and inverse design network based on the conditional variational autoencoder (Figure 3e). Glutamic acid enantiomers discrimination by a designed chiral metasurface was validated in CD angular differences.

## 3. AI-Assisted Various Biosensors

Biosensor signals are commonly disturbed easily by noise interference, signal lag, difficulty in analyzing high-dimensional data, and poor long-term stability. These issues severely limit the sensitivity, accuracy, and reliability of biosensing detection, particularly in complex biological samples and real-time monitoring scenarios. In contrast, the integration of artificial intelligence algorithms effectively addresses these bottlenecks by enabling intelligent noise reduction, dynamic signal compensation, and efficient feature extraction from high-dimensional datasets. Moreover, AI-assisted models enhance the long-term stability and adaptability of biosensors through continuous learning and self-calibration, thereby significantly improving the overall detection performance in terms of sensitivity, precision, and robustness, especially when applied to complex biological environments and real-time analytical tasks. This section will explore how AI can assist various biosensors (Table 2), including optical biosensors, electrochemical biosensors, microfluidics, and other (piezoelectric, thermal, mass, etc.) biosensors. Machine learning algorithms such as Random Forests, Support Vector Machines, and Linear Discriminant Analysis (LDA) are frequently employed to address high background noise and environmental interference in optical and electrochemical signals. For high-dimensional spectral signals from surface-enhanced Raman scattering (SERS) sensors and high-throughput data from microfluidics, CNN efficiently extract meaningful information. To meet practical application demands, RNN and Transformers enable real-time prediction and precise quantitative analysis, further enhancing response speed and detection sensitivity.

AI-assisted strategies effectively optimize the operational characteristics of diverse molecular biosensors, including optical, colorimetric, SERS, electrochemical, microfluidic, and piezoelectric platforms. Machine learning and deep learning algorithms enable automated data processing, intelligent signal denoising, spectral decomposition, dimensionality reduction, and nonlinear signal correction, which significantly improve sensitivity, detection limits, accuracy, anti-interference ability, and robustness in complex real-world environments. AI also supports multiplexed and rapid detection, reduces interference from experimental conditions, identifies key performance-related parameters, and enhances the reliability and stability of sensing systems. In microfluidic and wearable devices, AI improves droplet manipulation, cell sorting precision, and system durability, while for piezoelectric biosensors, it achieves high-accuracy classification and quantitative estimation. Collectively, artificial intelligence greatly elevates the practical analytical performance and application potential of molecular biosensors.

### 3.1. Optical Biosensors

Optical biosensors are a class of sensing devices that convert biomolecular recognition into measurable optical signals. Typically, an optical biosensor is composed of three core components: a biorecognition element (e.g., enzymes, antibodies, nucleic acids, or whole cells) immobilized on the surface of a transducer, an optical transducer (e.g., optical fiber, waveguide, interferometer, or surface plasmon resonance platform), and a signal detection and processing module. The working mechanism relies on specific binding between the biorecognition element and the target analyte, which induces changes in optical properties, including absorbance, fluorescence, refractive index, reflectivity, scattering, or luminescence [77]. These optical variations are then recorded, amplified, and processed to quantify the analyte. Owing to their high sensitivity, fast response, and label-free or label-based detection capabilities, optical biosensors can be applied to a wide range of analytes, such as small biomolecules (glucose, lactate, ions), proteins, antigens, antibodies, nucleic acids, pathogens, cancer biomarkers, and other biologically relevant species in environmental, clinical, and food samples [78]. The traditional optical biosensors face significant limitations. Colorimetric detection suffers from low accuracy, as subtle color changes are easily affected by environmental lighting and human visual differences [79,80]. Meanwhile, SERS analysis is hindered by spectral complexity, including nonlinearity, high-dimensionality, and overlapping peaks, that impedes clear interpretation and clinical translation [81]. Furthermore, conventional systems lack automated data processing, resulting in slower analysis and less robust multiplexed detection, which collectively reduces their reliability and real-world applicability. Above optical biosensor limitations resolved via strategies focus on device design, detection protocols, and classical signal processing. For colorimetric accuracy and SERS spectral complexity, methods include standardized lighting with spectrophotometers, optimized chromogenic conditions, improved SERS substrates, classical spectral preprocessing, and distinct Raman reporters [82,83]. Poor automation and multiplexing can be addressed by integrating microfluidic chips, array-based sensing, standardized data protocols, and optimized optical paths, effectively enhancing biosensor performance [84].

**Figure 4:**
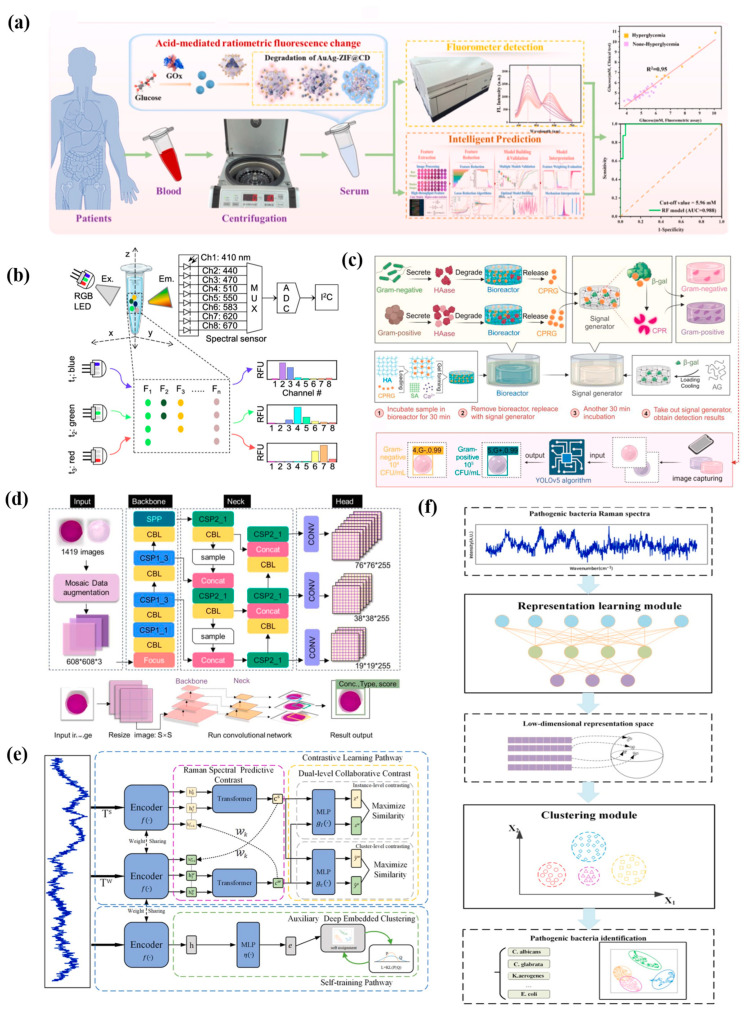
AI-assisted optical biosensors. (**a**) Blood glucose quantification from samples to intelligent prediction. Images reprinted from [63] with permission. (**b**) Multiple channels excited by an RGB LED light source. Images reprinted from [62] with permission. (**c**) Workflow of a colorimetric biosensor differentiated between Gram-positive and Gram-negative bacteria. (**d**) YOLOv5 model for accurate result output. Images reprinted from [64] with permission. (608*608*3 indicates feature dimensions.) (**e**) RamanCluster architecture. (**f**) Pathogenic bacteria were identified by Raman spectra with dimension reduction, feature extraction, and clustering. Images reprinted from [65] with permission.

In recent years, integration of AI technology with optical biosensors has gained significant attention for its potential in enhanced signal-to-noise ratios, automated data processing, minimization, and rapid multiplexed detection [85,86,87,88]. In a study by Chen et al. [63], an interpretable prediction algorithm was constructed for data processing automation, integrating ratiometric fluorescence biosensing. The machine learning pipeline comprised feature extraction, dimensionality reduction, prediction model construction, and model interpretation (Figure 4a). By automated data processing, the target concentration was determined in 5 s after image acquisition. Kshirsagar et al. [62] developed a multiplexed, lens-free, and universal fluorescence sensing platform using machine learning. Neural-network-based eight-channels detection showed better performance in concentration prediction and quantification limits than the traditional single-channel, due to complete spectral information, automatic compensation for nonlinear effects, and differentiation of signal cross-influence effects (Figure 4b). Accurate and rapid multiplexed detection via one-pot RT-LAMP underscored the robustness in real-word assay.

Colorimetric biosensors presented notable merits due to their simple manipulation, easy readout, and low price for on-site testing and real-time monitoring in a wide range of scenarios [89]. Nevertheless, the naked eye struggled to interpret colorimetric data with high accuracy, as minor color changes are susceptible to environmental elements like lighting circumstances and interindividual visual variations [90]. Leveraging AI technology can improve the accuracy of colorimetric detection, achieving rapid and precise data analysis [91]. Chen et al. [64] reported an AI-driven smartphone-based colorimetric biosensor detecting and differentiating bacteria with high sensitivity and accuracy. Hyaluronidase from bacteria reacted with chlorophenol red-β-D-galactopyranoside and β-galactosidase loaded in a hydrogel and then generated color in a concentration-dependent way (Figure 4c). The YOLOv5 algorithm for bacteria concentration output was constructed with three parts: a Cross-Stage Partial (CSP) network as backbone, three convolution layers as head, and neck connecting CSP with the Path Aggregation Network and the Spatial Pyramid Pooling (Figure 4d). The biosensor showed reliable performance in detecting *P. aeruginosa* or *S. aureus* in 60 min with an ultra-low limit of detection (LoD) of 10 CFU/mL.

SERS characterizes vibrational signatures of biomolecules with high specificity and sensitivity, enabling non-invasive and label-free sensing [92]. Advanced plasmonic probes strengthened SERS detection with enhanced stability, sensitivity and accuracy in complex biosamples [93]. However, spectral complexity, including non-linearity, high-dimensional data and overlapping peaks, hampered interpretation and clinical implementation [92,94]. Recently, incorporating AI into complex SERS spectrum analysis addressed the above challenges, thereby automating spectral decomposition, dimensionality reduction and signal-noise differentiation. Sun et al. [65] introduced an unsupervised Raman spectral identification method based on a novel deep clustering framework (RamanCluster) for pathogenic bacteria detection (Figure 4e,f). Without large labeled datasets, RamanCluster mapped the raw bacteria spectra by dimension reduction and accurately clustered various bacteria species. Moreover, RamanCluster showed robust performance in strong noise background or varying numbers of pathogenic bacterial species scenarios.

### 3.2. Electrochemical Biosensors

Electrochemical biosensors recognize biomolecules by biocatalytic reaction and affinity, with advantages of portability, rapid response, high sensitivity and specificity [95,96]. Specific binding between the biorecognition element and the target, which causes changes in the transducer’s electrical properties (e.g., current, voltage, impedance) that are measured and converted into quantitative analyte data. These biosensors can detect various analytes, including small biomolecules, proteins, nucleic acids, pathogens and biomarkers, and are widely used in clinical diagnosis, environmental monitoring and food safety. However, signal instability issues may still be a challenge in consideration of enzyme activity instability, weak specific recognition, material batch variations, electrode fouling, chemical interferences and so on [97,98]. Traditional strategies centered on optimizing biorecognition elements, electrode materials, and detection protocols offer an effective resolution to these issues [99]. Enzyme stability can be enhanced via immobilization in stable matrices and optimized reaction conditions, while specific recognition is strengthened by selecting high affinity biorecognition elements and pre-treating samples to reduce interference [100]. Additionally, standardizing material synthesis, modifying electrodes with anti-fouling materials, and implementing regular cleaning protocols address material batch variations and electrode fouling, ensuring stable signal transmission [101]. Recently, AI has been integrated with various electrochemical biosensors. Srivastava et al. [66] reported an electrochemiluminescence (ECL) sensor, detecting glucose, choline, and lactate with the assistance of the ML-based concentration prediction model. Seven regression models are trained and compared, and the Decision Tree model showed the best performance in R-squared scores. ML inclusion results in improved accuracy and stability, with linear ranges of 0.05–3 mM for Glucose, 0.1–4 mM for Lactate, and 0.0007–1 mM for Choline. Zhang et al. [67] reported a differential pulse voltammetry (DPV) biosensor detecting trace glucose, and utilized the ML method to diminish interference from experimental conditions. XGBoost model bypasses experimental conditions optimization in low-concentration glucose detection, while predicting concentration accurately under various test settings. Nonlinear issues brought by additive volumes and scan rates are interpreted and addressed by regression analysis. Uzun [68] trained 26 regression models for predicting and interpreting electrochemical biosensor responses. Variations, including the enzyme amount, crosslinker amount, glucose concentration and pH values, were defined as input features, and then fed into models from six methodological families. Gaussian Process Regression and wide artificial neural networks outperformed in accurate prediction and generalization. In addition, by SHAP analysis, the framework interpreted that enzyme amount, pH, and analyte concentration are the most important factors to performance, providing guidance to accurate, reproducible and robust sensor design.

Algorithms were widely used in multi-modal data processing, significantly enhancing accuracy, selectivity and sensitivity with informed decision. Kammarchedu et al. [69] fabricated a wearable eMoSx-LIG sensor for detecting tyrosine and uric acid in mixed samples, and conducted multimodal electrochemical sensing with an ML framework. The raw cyclic voltammetry, square wave voltammetry, differential pulse voltammetry, and large amplitude AC voltammetry data are decomposed, fit with constraints, and finally used for model training. The optimized multimodal model architecture enabled a twofold improvement in LoD, compared with the conventional single signal method. In addition, the printed eMoSx-LIG flexible sensor was applied in measuring tyrosine and uric acid on-body sweat.

### 3.3. Microfluidics

**Figure 5:**
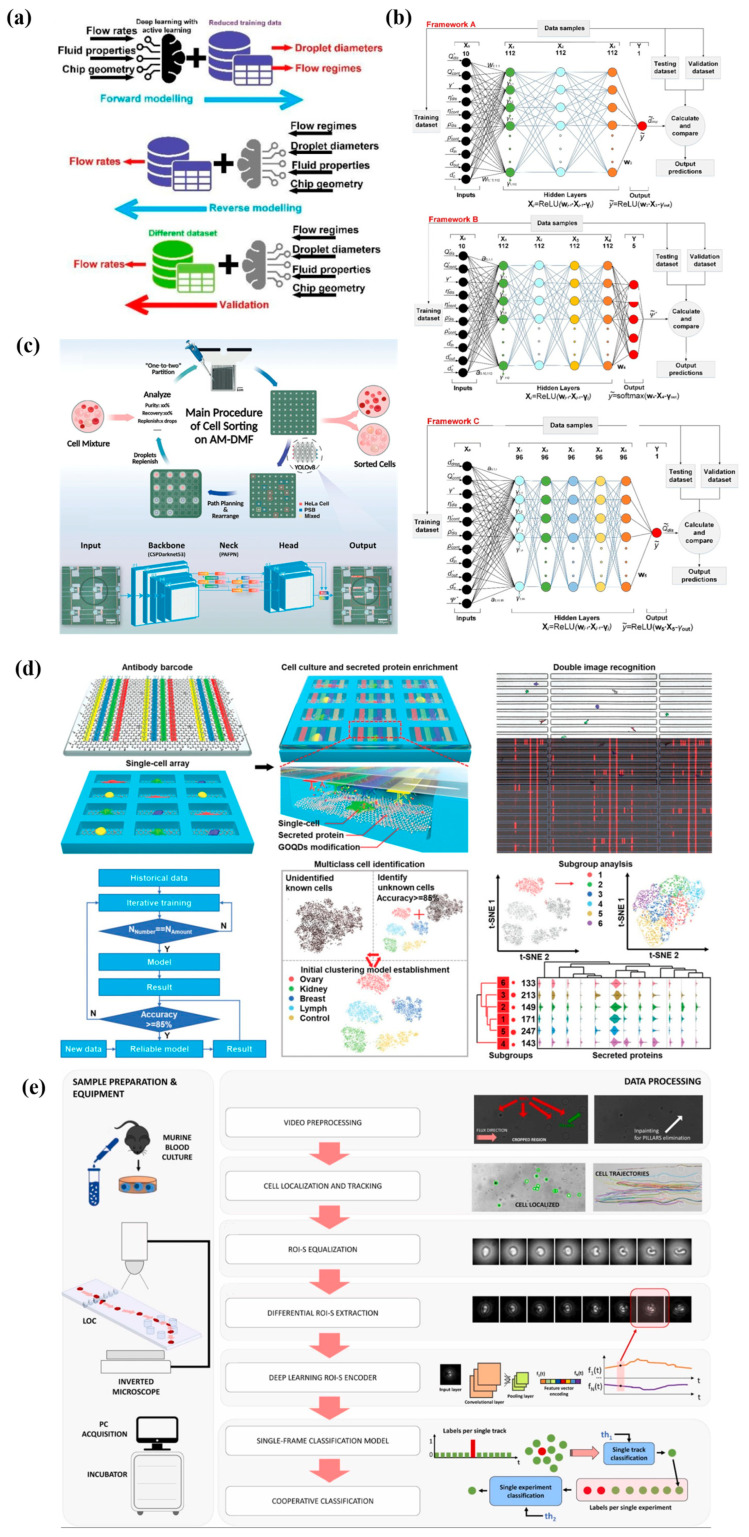
AI-assisted microfluidic biosensors. (**a**) Forward and reverse modeling for ideal droplets. (**b**) Framework A, B, and C for droplet diameter, flow regime, and volumetric flow rate predictions. Images reprinted from [71] with permission. (**c**) Digital microfluidic chips for cell sorting assisted by deep learning models. Images reprinted from [72] with permission. (**d**) Machine learning assisted living cell secretion profiling microfluidic chips. Images reprinted from [73] with permission. (**e**) Blood disease diagnosis with a deep learning encoder and a single-frame classification model. Images reprinted from [74] with permission.

Within microfluidic chips, microliters of liquid flow simultaneously through multiple micrometer-sized channels or chambers to different reaction zones by manipulation, detecting molecules and then releasing optical or electrical signals [102]. Microfluidic biosensors have the advantages of miniaturization, reduced sample and reagent consumption, high reaction efficiency, high throughput, and precise fluid controllability [103]. Therefore, microfluidics shows great potential in drug screening, diagnosis, cell biology, environment and food safety monitoring [104,105,106,107]. AI accelerates microfluidic systems in data processing and decision making with an automatic and accurate manner. Liang et al. [70] reported deep reinforcement learning, addressing the reliability issue caused by electrode degradation in digital microfluidic biochips. The dynamic status of aged electrodes is learned, and then the framework designs new paths to avoid unhealthy electrodes. Droplet routing assisted by deep learning enables reliable droplet manipulation, saves reagent and extends microfluidic biochip lifespan. In addition, deep learning assists droplet generation with desired size, density and dispersity. Wang et al. [71] reported a dual-directional deep learning model predicting droplet diameters and flow regimes based on flow rates, fluid properties and chip geometry by active learning in a small-scale training dataset (Figure 5a). Three DNN frameworks, marked as frameworks A, B, and C, predict droplet diameter, flow regime and volumetric flow rates separately (Figure 5b). Framework C calculates a droplet diameter first based on control parameters and material properties, inputs the diameter to frameworks A and B, and then obtains the final estimated diameter by combining the results from A and B. The dual-directional modeling method added framework B and C shows superior predictive performance with 9.90% relative error, increased by 45.6% compared to the single-direction method. Another work [72] combined deep learning algorithms with droplet microfluidics for automated droplet manipulation and label-free cell sorting based on morphology feature (Figure 5c). YOLOv8 model outperforms v7 and v5 in precision and runtime, and thereby trains with various cell mixture datasets. The deep learning assisted cell sorting platform presents notable performance in precise isolation from complex mixtures, cell purity over 96% and 80% recovery rate.

Machine learning is inspired by the human brain, helps microfluidic biosensors’ data process by regression, classification and clustering, and acquires new conclusions automatically. For example, Wang et al. [73] developed a high-throughput living cell secretion profiling microfluidic chip, powered by a K-means strategy for biomarker analysis and tumor cell classification (Figure 5d). Graphene oxide quantum dots assemble an antibody of biochip-separated cells and label proteins with fluorescence. Graphene oxide quantum dots are self-assembled on the antibody barcode chip for the detection of secreted biomarkers from isolated single cells. Unsupervised clustering method classifies 5286 single cells into five types with 95% average accuracy. By normalization, each cell type is characterized with representative biomarkers: HEY cells with high OPN and IL8 expression level, MDA-MB-231 cells with high IL-6 expression level, 293T cells with high HSP70 expression, but K562 cells with low Mesothelin and IL-8 expression. Mencattini et al. [74] proposed microfluidics integrated with machine learning, monitoring red blood cell and evaluating cell plasticity in Pyruvate Kinase Disease samples (Figure 5e). The chip is designed with forest pillars, imitating blood cells passing through the spleen’s reticular structure. When cells are pumped into and flow through pillars, videos are acquired by time-lapse microscopy for offline analysis. Features are extracted by transfer—learning method, and classified cells’ health states by track-level and experiment-level strategies. Both the AlexNet-based method with a single threshold and NasNetLarge method with two thresholds demonstrate the highest accuracy of 85%.

### 3.4. Other Biosensors

Aside from common optical biosensors, electrochemical sensors, and microfluidic sensors, there are also piezoelectric, thermal, and mass biosensors. Piezoelectric biosensors operate based on the piezoelectric effect. When detecting biomolecules, forces applied to the piezoelectric material’s surface cause changes that alter its resonant frequency, resulting in an electrical signal output. Due to interference from multiple components, environmental variations, and non-specific adsorption effects, traditional methods struggle to effectively distinguish genuine signals from interference within weak, dynamic response curves. AI enables robust extraction and classification of weak sensing signals, thereby significantly improving the accuracy of signal identification in biosensors. Furthermore, through advanced neural network models, it processes complex sensing data to achieve precise quantitative analysis and reliable target recognition. Wu et al. [75] presented a deep learning model, composed of a CNN and a Bidirectional Long Short-Term Memory network (BiLSTM), enabling robust extraction and classification of the respiratory monitoring signal. Assisted by the CNN-BiLSTM hybrid model, the piezoelectric-ferroelectret-based respiration monitor show 100% accuracy in classifying weak, normal, and coughing states. Hashem et al. [76] reported a piezoelectric sensor for tumor detection, sensing tissue localization and stiffness in a non-invasive way. Recurrent neural networks and feedforward neural networks are utilized in processing absorber frequencies into tumor size, location, and stiffness. High accuracy in tumor size and Young’s modulus estimation is verified with a minimum error of 0.04 mm and 0.0319 kPa, which reinforces the sensor’s potential in early diagnosis and tumor monitoring.

Thermal biosensors are based on the measurement of heat changes (enthalpy variations) generated or absorbed during biorecognition reactions (e.g., enzymatic catalysis, antibody–antigen binding). By integrating a thermistor or a thermopile as a transducer, they convert temperature variations into measurable electrical signals [108]. Mass biosensors are represented mainly by quartz crystal microbalance, and surface acoustic wave devices, monitoring the mass change adsorbed on the sensitive interface via shifts in resonant frequency, according to the Sauerbrey equation [109]. They are label-free platforms suitable for the real-time monitoring of biomolecular interactions, including DNA hybridization, protein–protein binding, and whole-cell detection. However, both thermal and mass biosensors have obvious limitations that severely restrict their extensive research and practical application [110,111]. Thermal biosensors are characterized by a rather complex structure and control system, mainly because the extremely weak heat generated by biochemical reactions requires precise thermostatting devices to eliminate environmental temperature fluctuations, making the whole system difficult to simplify and integrate. They also possess low sensitivity due to the small amount of heat released by most biorecognition reactions and the easy dissipation and loss of heat, which hinders the accurate detection of low-concentration targets. In addition, their detection specificity is unsatisfactory due to significant non-specific heating effects, as environmental changes, solvent mixing, impurity reactions, and other interferences can produce background heat that is hard to distinguish from the effective signal of target bioreactions, reducing detection reliability. Similarly, mass biosensors are highly vulnerable to non-specific adsorption. Impurity proteins, small molecules, and contaminants in complex samples can non-specifically bind to the sensing interface, causing signal drift, high false-positive rates, and poor reproducibility. They are also extremely sensitive to the detection environment, as slight fluctuations in liquid viscosity, density, temperature, and flow rate can significantly affect the resonance signal and lead to insufficient system stability. Furthermore, it is difficult to balance their sensitivity and selectivity—improving sensitivity usually amplifies environmental and impurity interferences, greatly restricting their application in complex systems such as real body fluids and clinical samples. Though, we found few research integrated AI with thermal and mass biosensor. It is hoped that AI could help both types of sensors break through their bottlenecks in the future, promote their extensive research and application, and further improve their overall performance. Specifically, AI may provide intelligent guidance for optimizing sensor structure and bioreceptor design, while also enhancing the accuracy and efficiency of signal analysis and interference filtering during data processing.

## 4. Monitoring by a Wearable AI-Based Biosensor

Biosensors integrated with flexible electronics convert small-molecule detection into electrical signals in real time, enabling precise monitoring. Emerging developments in real-time monitoring—such as wearable biosensors, implantable biosensors, and smartphone-assisted biosensors—offer advantages including wireless connectivity, remote operation, device miniaturization, non-invasiveness, and rapid response [112,113,114,115]. Real-time biosensor monitoring is revolutionizing medical diagnostics, environmental management, and food safety [116,117,118]. However, practical implementation still encounters multiple challenges, including signal interference from complex environments, efficient and timely processing of massive data volumes, and limited sensor lifespan.

In recent years, AI has attracted increasing attention in biosensor data processing, overcoming limitations of noise reference, irregular signals, poor stability and delayed readout [119]. Algorithms for calibration and predicting enabled sensing platforms to accurately detect on-site molecular concentrations and predict real-time target analytes, which was instrumental in fields of food safety, healthcare, and environmental monitoring [120,121]. Furthermore, connecting portable devices, Internet of Things (IoT), and AI-based biosensors notably facilitated real-time monitoring, for wirelessly transmitted data and timely readout [122].

### 4.1. Real-Time Wearable Monitoring

Wearable devices display significant advantages in real-time monitoring, characterized by portability, lightweight design, long-term wearability, low cost, non-invasiveness, and rapid response times. They enable continuous, dynamic collection of physiological and biochemical information without interrupting daily life or physical activity, holding substantial importance for personal health management, chronic disease monitoring, exercise science, critical care, and telemedicine [123,124,125]. Signal molecules detected by wearable devices primarily include glucose, lactate, alcohol, uric acid, potassium and sodium ions, cortisol, and various sweat metabolites [126]. These analytes are characterized by low concentrations, susceptibility to environmental and physiological interference, poor stability in surface samples, and complex background components [99,127,128]. Consequently, biosensors face challenges such as high noise interference, significant baseline drift, response delays, and reduced accuracy during real-time monitoring [129,130]. Therefore, integrating AI technology with wearable molecular sensors can significantly enhance the system’s anti-interference capability, detection accuracy, and response speed through signal denoising, feature extraction, model calibration, and prediction algorithm optimization [119,131,132,133]. This enables stable and reliable performance during prolonged dynamic monitoring, thereby effectively advancing the practical application and industrial development of wearable biosensors.

**Figure 6:**
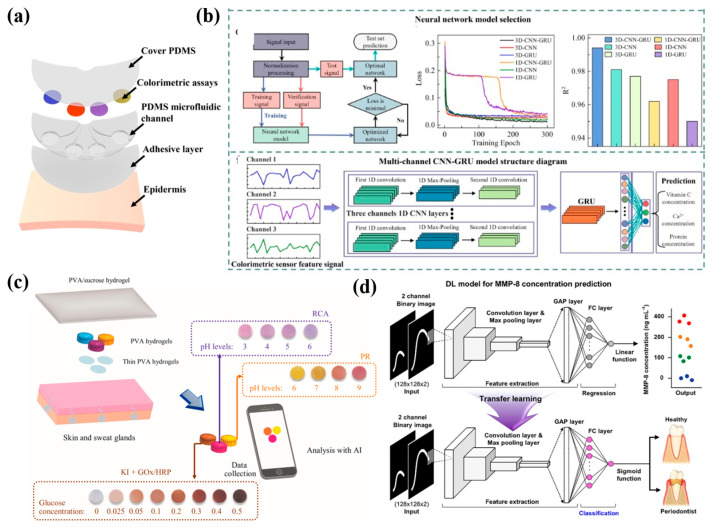
Real-time monitoring of wearable biosensors. (**a**) The structure of a microfluidic colorimetric biosensor. (**b**) Flowcharts of selected models and model structure diagrams. Images reprinted from [134] with permission. (**c**) A composite hydrogel patch to monitor pH and glucose values in the sweat. Images reprinted from [135] with permission. (**d**) DL model for MMP-8 concentration prediction. Images reprinted from [136] with permission.

A deep learning assisted microfluidic colorimetric biosensor was proposed to monitor vitamin C, pH, Ca^2+^, and proteins in tears by a smartphone-based cloud server data analysis system (Figure 6a) [134]. A three-channel convolutional recurrent neural network is the optimized model with the highest accuracy in predicting four biomarker concentrations, correcting errors from variable pH value and color temperature. CNN specializes in extracting spatio-temporal features, while GRU handles long sequences and variable-length data effectively. Combining the two not only enables more comprehensive modeling of sequence information but also adapts to scenarios where signal data lengths vary (Figure 6b).Chenaini et al. [135] developed a bilayer hydrogel-based wearable patch for continuous real-time monitoring of sweat pH and glucose levels, and employed RF and CNN models for accurate sweat analysis. CNN demonstrates robust high-dimensional feature learning capabilities, directly extracting key features from raw data while capturing spatial distribution variations. It achieves optimal performance in glucose concentration prediction (R^2^ = 0.988). RF excels in rapid training and straightforward deployment, adept at handling complex nonlinear relationships. It exhibits exceptional stability and outstanding fitting performance (~0.99) in pH prediction across varying acidity environments. Jeon et al. [136] integrated deep learning with a wearable sensor, detecting inflammation biomarkers in saliva and monitoring oral health. This work constructs a specialized CNN deep learning architecture for MMP-8 biomarker concentration regression. Feature extraction and refinement are achieved through convolutional layers, max pooling, global average pooling, and fully connected layers. Combined with transfer learning, it enables binary classification diagnosis of periodontitis health versus disease (Figure 6d). The deep learning model achieves precise quantitative prediction across the clinical concentration range of 0–400 ng mL^−1^. Transfer learning effectively mitigates the challenges posed by limited clinical data, thereby enhancing diagnostic accuracy.

### 4.2. Low Power Consumption

The energy consumption of wearable biosensors primarily stems from continuous sensor data acquisition, analog-to-digital conversion, wireless transmission, and backend data processing [137]. High energy consumption directly limits device battery life, miniaturization design, and long-term wear comfort, severely impacting the continuity of health monitoring and user compliance [138,139,140]. Reducing energy consumption is crucial for achieving long-term continuous monitoring, flexible miniaturization, and self-powered operation, which is the core prerequisite for wearable devices to transition from experimental use to clinical adoption. Researchers have optimized more intelligent algorithms that reduce computational power, memory, and data transmission consumption while maintaining detection accuracy [119,141].

Farhana [142] proposed applying Physical Information Neural Networks (PINNs) to edge carbon nanotube biosensors, embedding physical partial differential equations to construct an efficient edge intelligent detection solution. By integrating the physical principles of biosensors during training, PINNs can learn true signal features with fewer data points and shallower networks. This approach ensures high-precision predictions while reducing data requirements, alleviating computational burdens, and ultimately lowering device energy consumption. Compared to traditional convolutional neural networks, this model achieves 92.8% classification accuracy while reducing power consumption by 23.8%. This approach effectively alleviates energy constraints at the model level for edge wearable biosensing applications. Baghersalimi et al. [143] introduced the Multi-to-Single Knowledge Distillation (M2SKD) strategy, which simplifies signal requirements and reduces sensor and data dependencies to achieve low-power, compact, and highly comfortable long-term wearable monitoring. M2SKD first trains a high-precision yet computationally complex model using multiple biosignals, fully leveraging multimodal information to acquire reliable epilepsy detection knowledge. Through knowledge distillation, it then transfers the learned decision patterns to a model using only a single biosignal. This approach maintains near-original accuracy while reducing sensor and computational requirements. Zhao et al. [144] presented the lightweight spiking neural network (LSNNet) for edge devices, enabling depression state recognition with low power consumption. LSNNet adopts a lightweight spiking neural network (SNN) architecture that leverages event-driven temporal computation. It performs operations only when signals change, reducing redundant computations and energy consumption at the fundamental level. By minimizing parameters, FLOPs, and memory usage, it achieves model lightweighting, significantly reducing computational load and power consumption on microcontrollers. This enables adaptation to resource-constrained wearable hardware.

### 4.3. Robustness and Reliability

Wearable sensors are susceptible to environmental interference, contamination, light variations, and cross-interference from physiological components in real-world dynamic wear environments [145,146]. These factors cause signal drift and detection errors, making it difficult to ensure stable output. Traditional sensing methods rely on single-signal calibration and lack adaptive correction capabilities for complex interference, resulting in significantly reduced reliability and robustness in real-world scenarios. Artificial intelligence effectively suppresses signal shifts caused by environmental factors and motion by learning multidimensional interference characteristics and establishing adaptive correction models [147,148,149]. Machine learning can also extract stable correlation patterns from large amounts of actual measurement data, achieving interference rejection and signal fidelity, thereby enhancing system stability at the algorithmic level [150,151,152].

**Figure 7:**
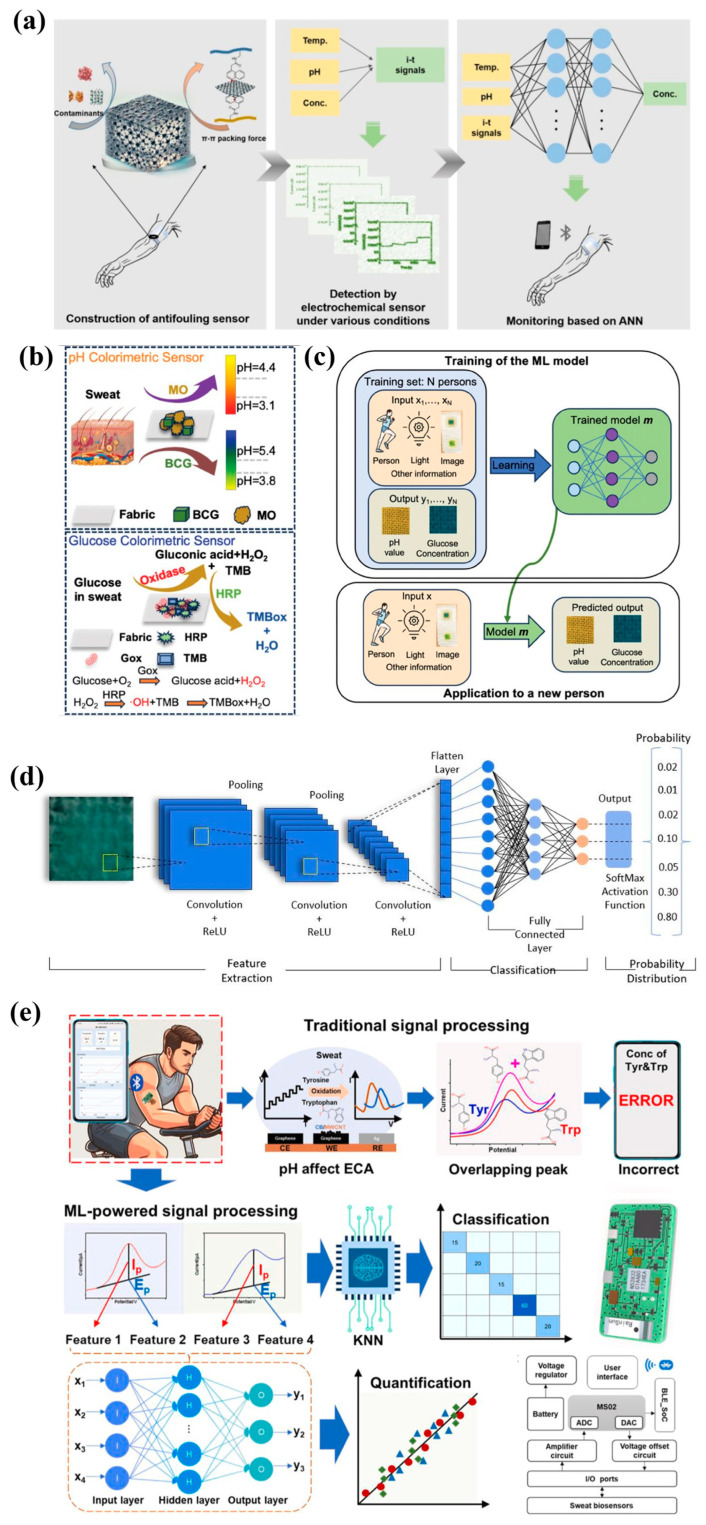
Robust and reliable AI—wearable biosensors. (**a**) ANN assisted sensor monitoring of dynamic physiological conditions. Images reprinted from [153] with permission. (**b**) pH and glucose sensors. (**c**) Trained machine learning models for pH and glucose concentration prediction. (**d**) CNN model, as one of three models utilized to extract features and classify data. Images reprinted from [154] with permission. (**e**) Machine learning powered sweat sensor solves challenges caused by overlapping oxidation peaks and pH interference. Images reprinted from [155] with permission.

Lv et al. [153] developed a wearable sweat-sensing system with anti-contamination properties tailored for dynamic wear scenarios. Leveraging machine learning, it achieves stable uric acid detection under complex conditions. The artificial neural network (ANN) effectively separates environmental interference from target signals by learning experimental data across multiple dynamic physiological conditions (Figure 7a). This approach overcomes the limitations of traditional sensors optimized for single variables, enhancing the sensor’s anti-interference capability. This ANN model demonstrated outstanding performance in real sweat validation, enabling highly accurate uric acid prediction (R^2^ = 0.9989) while effectively resisting impacts from biological contamination and dynamic variations, significantly enhancing the biosensor’s robustness. Zhou et al. [154] developed a battery-free, low-cost colorimetric wearable biosensor based on cotton textiles, integrating machine learning for non-invasive continuous detection of target indicators in sweat (Figure 7b,c). AI analyzed collected sensor images using three distinct machine learning algorithms (Figure 7d). Combined with image processing techniques, it filtered out interference factors, corrected color signal deviations, and enhanced the sensor’s resistance to external disturbances, thereby improving its robustness. The integration of LDA and SVM enabled the sensor to achieve 90% accuracy in detecting pH values between 4 and 10 and glucose concentrations ranging from 0.03 to 1 mM. This effectively mitigated human interpretation errors while strengthening the sensor’s detection stability and interference resistance, ultimately enhancing overall robustness. Zhou et al. [155] proposed an intelligent wearable sensing interface that leverages machine learning for precise differentiation and reliable prediction of multi-component sweat sensors. The k-nearest neighbor model utilizes four features to distinguish different mixed data types, while the backpropagation neural network model learns complex nonlinear mappings between sensor electrical signals and biochemical indicators, thereby resolving detection challenges caused by overlapping oxidation peaks and pH interference in mixed solutions (Figure 7e). This wearable sensor rapidly and accurately classifies mixed components while simultaneously quantifying tyrosine, tryptophan, and pH levels, providing a reliable solution for precise sensing in complex bodily fluid environments.

### 4.4. Personalized and Self-Adaptive Function

Artificial intelligence significantly enhances the personalization and adaptive capabilities of sensing systems. AI models can be customized for specific application scenarios, demonstrating exceptional performance in early disease diagnosis, health monitoring, and therapeutic efficacy assessment within the medical field. Furthermore, AI-driven sensors with adaptive capabilities can intelligently monitor pollutants across diverse environments. AI-based wearable sensors dynamically adjust to individual variations and operational conditions, thereby enhancing detection accuracy and stability, laying a solid foundation for reliable real-world applications. Nazir et al. [156] developed a paper sensor monitoring water toxicity, quantified by customized AI software Scentinel. Variation brought by smartphone CMOS limited sensitivity and accuracy, which was addressed by on-board calibration. The affordable all-in-one sensor showed promising performance in both tap water and wastewater onsite testing, equipped with an AI-powered application for labeling and extracting signal value. In the healthcare area, real-time monitoring facilitated early diagnosis, personalized treatment and informed clinic decisions. Rabby et al. [157] proposed a deep RNN model for blood glucose prediction, continuously monitoring diabetes patients’ glucose levels. Multiple types of features, such as glucose level, step count, carbohydrate intake from the meal, and bolus dose, were fed into an RNN model with stacked LSTM model training, which outputs the highest prediction accuracy.

## 5. Conclusions and Outlooks

Although significant progress has been made in integrating AI with molecular wearable biosensors, there are still some challenges. At the molecular level, biosensing signals often arise from complex nonlinear biochemical interactions, including multi-valent binding, competitive adsorption, and dynamic conformational changes of recognition elements. Mainly based on statistical signal pattern training, AI models may unintentionally capture correlations but cannot reflect the underlying molecular mechanisms. This mismatch raises concerns about mechanism interpretability and limits the ability to convert model predictions into molecular insights.

In the usage scenarios of wearable devices, challenges related to data further increase the complexity of AI deployment. Continuous monitoring generates a large amount of time-dependent data, but high-quality labeled data on biochemical events is still scarce. The lack of purified training datasets adds to the uncertainty. Individual differences, environmental fluctuations, and differences between devices introduce more uncertainty, making the generalization of the model difficult. Context-based molecular baselines require adaptive learning strategies, but this personalization increases the complexity of the model and computational requirements.

Also, from a systems perspective, energy limitations and hardware limitations re-strict the implementation of computationally intensive algorithms on wearable platforms. Balancing model complexity and energy efficiency remains a core engineering challenge. How to get a long-term monitoring device remains a challenge. Additionally, the interpretability and regulatory acceptability of AI-driven biosensing systems need careful consideration, especially in clinical applications, where transparent decision-making is crucial.

The cost and personnel requirements of AI application are key concerns widely acknowledged in the field. The widespread availability of open-source large language models and AI frameworks has significantly lowered the threshold and cost of AI applications in biosensors. Pre-trained models can be adopted, and lightweight AI models can run efficiently on low-cost hardware, thus reducing expenses related to data annotation, model optimization and hardware deployment. Meanwhile, AI-integrated biosensors feature high stability and user-friendly interfaces. Staff who have basic operational training are able to complete model calibration, data collection and result interpretation, which greatly lowers the professional barrier for the practical application and popularization of AI-assisted biosensors.

Therefore, future research should focus on mechanism-based AI model construction, standardized datasets for wearable molecular sensing, lightweight and interpretable algorithms, and closed-loop integration of molecular recognition, signal transduction, and intelligent analysis. Solving these challenges is crucial for transforming AI-assisted molecular biosensors from experimental prototypes to reliable and practical healthcare solutions.

## Figures and Tables

**Table 1:** Comparison of different AI algorithms.

Category	Specific Examples	Core Ideas	Method Characteristics
Machine Learning	Support Vector Machine (SVM) [16]	Its core is to partition data of different categories by finding the optimal separating hyperplane.	Feature extraction requires manual construction based on specific domain knowledge, which brings strong interpretability. Nevertheless, the cost of feature extraction is relatively high, demanding considerable time and effort to ensure its effectiveness.
	Random Forest [17]	Based on the idea of ensemble learning, it reduces the risk of overfitting by constructing multiple decision trees and fusing their prediction results.	
Deep Learning	Convolutional Neural Network (CNN) [18]	With convolution operation as the core, it automatically extracts data features through convolutional and pooling layers.	Features are automatically extracted by deep learning networks, and the extracted features are mostly abstract with weak interpretability. The cost of feature extraction is low, which saves labor costs, yet a large volume of data is required for training to guarantee the accuracy of feature extraction.
	Transformer [19]	Based on the self-attention mechanism, it can capture long-range dependency relationships in data.	
	Generative Adversarial Network (GAN) [20]	It consists of a generator and a discriminator that conduct adversarial training with each other; the generator is responsible for generating samples consistent with the real data distribution, while the discriminator distinguishes between real and generated samples.	
	Recurrent Neural Network (RNN) [21]	Designed for sequential data, it can memorize historical information and is applicable to tasks such as time series prediction and natural language processing.	

**Table 2:** Comparison of AI-assisted various biosensors.

Type	Algorithm	Analyte	Analytical Characteristics	Description	Refs.
Optical biosensors	Multivariate Linear Regression Support Vector Regression Neural Network	respiratory viruses	LoD: 0.01 μM	a multiplexed, lens-free, and universal fluorescence sensing platform using machine learning	[62]
	Random forest algorithm	glucose	LoD: 50.9 μM	an interpretable prediction algorithm, integrating ratiometric fluorescence biosensing	[63]
	YOLOv5 algorithm	pathogenic bacteria	LoD: 10 CFU/mL	AI-driven smartphone-based colorimetric biosensor, detecting and differentiating bacteria with high sensitivity and accuracy	[64]
	RamanCluster	pathogenic bacteria	-	robust performance in strong noise background or varying numbers of pathogenic bacterial species scenarios.	[65]
Electrochemical biosensors	Huber, Random Sample Consensus, Theil-Sen, Support Vector Machine Regression, K-Nearest Neighbor, Decision Tree, and Random Forest	glucose, choline, and lactate	LoD: 0.033 mM, 0.07 mM, 0.001 mM	an electrochemiluminescence sensor, detecting glucose, choline, and lactate with assistance of ML-based concentration prediction model.	[66]
	Multiple Linear Regression, Decision Trees, Artificial Neural Networks, Random Forest, and Extreme Gradient Boosting	glucose	Mean Absolute Error (MAE) = 21.6%, Root Mean Square Error (RMSE) = 25.3%	a differential pulse voltammetry biosensor detecting trace glucose, and utilized ML method to diminish interference	[67]
	linear, tree-based, kernel-based, Gaussian process, artificial neural networks, and stacked ensembles	glucose	RMSE = 0.143	framework interpreted that enzyme amount, pH, and analyte concentration as the most important factors to performance	[68]
	decision tree algorithm	tyrosine uric acid	LoD: 100 nM 10 nM	multimodal electrochemical sensing with ML framework	[69]
Microfluidics	deep reinforcement learning	Chromatin Immunoprecipitation	-	deep reinforcement learning, addressing reliability issue caused by electrode degradation	[70]
	dual-directional deep learning active learning algorithm	droplet generation	-	a dual-directional deep learning model predicting droplet diameters and flow regimes based on flow rates, fluid properties and chip geometry by active learning	[71]
	YOLOv8 model	cell sorting	98.5% sorting precision, 96.49% purity, and an 80% recovery	deep learning algorithms combined with microfluidics for automated droplet manipulation and label-free cell sorting based on morphology feature	[72]
	K-means algorithm	proteins	95.0% recognition accuracy	high-throughput living cell secretion profiling microfluidic chip, powered by K-means strategy for biomarker analysis and tumor cell classification	[73]
	AlexNet, ResNet101, and NasNetLarge	red blood cell	over 85% accuracy	microfluidics integrated with machine learning, monitoring red blood cell and evaluating cell plasticity in Pyruvate Kinase Disease samples	[74]
Piezoelectric biosensors	CNN and a Bidirectional Long Short-Term Memory network	respiration monitoring	100% classification accuracy	a piezoelectric-ferroelectret-based respiration monitor show 100% accuracy in classifying weak, normal, and coughing states	[75]
	Recurrent neural networks and feedforward neural networks	tumor detection	a minimum error of 0.04 mm in tumor size estimation, 0.0319 kPa in stiffness detection	a piezoelectric sensor for tumor detection, sensing tissue localization and stiffness in a non-invasive way	[76]

## Data Availability

The original contributions presented in this study are included in the article. Further inquiries can be directed to the corresponding authors.

## Abbreviations

The following abbreviations are used in this manuscript:

AI	Artificial intelligence
ADT	Androgen deprivation therapy
GPN	Geometry-predicting-network
SPN	Spectrum-predicting-network
CD	Circular dichroism
RF	Random Forests
SVM	Support Vector Machines
LDA	Linear Discriminant Analysis
SERS	Surface-enhanced Raman scattering
CNN	Convolutional Neural Networks
RNN	Recurrent Neural Networks
CSP	Cross-Stage Partial
LoD	Limit of detection
ECL	Electrochemiluminescence
DPV	Differential pulse voltammetry
BiLSTM	Bidirectional Long Short-Term Memory network
IoT	Internet of Things
PINNs	Physical Information Neural Networks
M2SKD	Multi-to-Single Knowledge Distillation
LSNNet	Lightweight spiking neural network
SNN	Spiking neural network
ANN	Artificial neural network
